# Editorial for the Special Issue “Innovation in Oral- and Cranio-Maxillofacial Reconstruction”

**DOI:** 10.3390/cmtr19030036

**Published:** 2026-08-19

**Authors:** Susana Heredero-Jung, Richard Yuxiong Su, Satyesh Parmar

**Affiliations:** 1 Oral and Maxillofacial Surgery Department, University Hospital Reina Sofía (HURS), E-14004 Cordoba, Spain; 2 Division of Oral and Maxillofacial Surgery, Faculty of Dentistry, University of Hong Kong, Hong Kong, China; 3 Department of Oral and Maxillofacial Surgery, University Hospital of Birmingham, Birmingham B15 2WB, UK

## Advancements and Future Directions in Cranio-Maxillofacial Reconstruction: Embracing Innovation to Improve Patient Outcomes

We are delighted to present this Special Issue on Innovations in Oral and Cranio-Maxillofacial Reconstruction, which collects a broad range of contemporary research and emerging technologies shaping the future of reconstructive surgery and improving patient care.

Traditional reconstructive surgery, which depends heavily on surgeons’ experiences, manual templating, and off-the-shelf titanium hardware, is shifting to a patient-specific, data-driven, and biologically informed approach. This issue highlights several important advances, including the use of 3D-printed PEEK implants and the application of reconstruction plates in the management of atrophic mandibles. It also features comparative studies evaluating hybrid arch bar systems alongside the growing role of 3D facial scanning in surgical planning and assessment.

Digital innovation is a recurring theme throughout the issue, with articles describing automated computer-assisted methods for determining optimal dental implant positioning and fibular flap dimensions for jaw reconstruction. Algorithms can now analyze patients’ CT data and automatically propose the ideal position for dental implants in fibula flaps, aligning prosthetic needs with available bone volume and occlusal relationships, thereby reducing the pre-operative planning burden and minimizing trial and error. Readers will also find contributions on advances in genioplasty and the development of MRI-only workflows for mandibular resection planning, reflecting the continuing evolution of image-guided surgery.

In addition, this issue includes studies addressing maxillofacial trauma, tissue engineering, facial contouring, and zygomatic implant-perforated flaps. Together, these articles demonstrate the breadth of innovation currently transforming oral and cranio-maxillofacial reconstruction, ranging from digital technologies and biomaterials to refined surgical techniques and enhanced patient pathways.

The future of oral and cranio-maxillofacial reconstruction is promising. The field is moving toward more precise and reliable surgical techniques, with the use of patient-specific computer models and 3D imaging to plan and guide surgeries, helping reduce complications, improve healing, and restore function more effectively. We can anticipate the routine integration of artificial intelligence/machine learning not only for planning but also for intraoperative navigation, where real-time AI can adjust surgical trajectories on the basis of subtle tissue shifts or unexpected anatomical variations, effectively acting as a co-pilot for surgeons. As technology continues to evolve, surgeons are becoming better equipped with the skills and tools needed to deliver personalized care. The ultimate goal is to improve quality of life for patients by making reconstructive procedures safer, more predictable, and more effective. Additionally, advances in our understanding of the biological and regenerative aspects of healing are guiding us toward more effective, minimally invasive procedures. We are seeing a shift towards using biomaterials that respond to the body’s natural conditions to improve outcomes. In parallel, innovations in 4D bioprinting, which involves creating structures with materials that change over time, are introducing new possibilities. These developments allow us to design custom scaffolds that better match a patient’s anatomy and promote proper bone growth, although the clinical applications may still require further validation.

We hope this Special Issue provides valuable insights for clinicians, researchers, and trainees alike and stimulates further research and collaboration in this rapidly advancing field. We are grateful to the authors, reviewers, and editorial team, whose expertise and dedication made this collection possible, and we trust that readers will find it both informative and inspiring.

## Data Availability

No new data were created or analyzed in this study. Data sharing is not applicable to this article.

